# *In vivo* evaluation of [^18^F]fluoroetanidazole as a new marker for imaging tumour hypoxia with positron emission tomography

**DOI:** 10.1038/sj.bjc.6601862

**Published:** 2004-05-11

**Authors:** H Barthel, H Wilson, D R Collingridge, G Brown, S Osman, S K Luthra, F Brady, P Workman, P M Price, E O Aboagye

**Affiliations:** 1 Cancer Research UK PET Oncology Group, Department of Cancer Medicine, Faculty of Medicine, Imperial College London, Hammersmith Hospital Campus, Du Cane Road, W12 0NN London, UK; 2 Hammersmith Imanet, Cyclotron Building, Hammersmith Hospital, Du Cane Road, W12 0NN London, UK; 3 Cancer Research UK Centre for Cancer Therapeutics, The Institute for Cancer Research, Brookes Lawley Building, Room 0E8, 15 Cotswold Road, Sutton, Surrey SM2 5NG, UK; 4 Department of Nuclear Medicine, University of Leipzig, Liebigstraße 20A, 04103 Leipzig, Germany

**Keywords:** [^18^F]FETA, PET, tumour hypoxia, 2-nitroimidazole, imaging

## Abstract

Development of hypoxia-targeted therapies has stimulated the search for clinically applicable noninvasive markers of tumour hypoxia. Here, we describe the validation of [^18^F]fluoroetanidazole ([^18^F]FETA) as a tumour hypoxia marker by positron emission tomography (PET). Cellular transport and retention of [^18^F]FETA were determined *in vitro* under air *vs* nitrogen. Biodistribution and metabolism of the radiotracer were determined in mice bearing MCF-7, RIF-1, EMT6, HT1080/26.6, and HT1080/1-3C xenografts. Dynamic PET imaging was performed on a dedicated small animal scanner. [^18^F]FETA, with an octanol–water partition coefficient of 0.16±0.01, was selectively retained by RIF-1 cells under hypoxia compared to air (3.4- to 4.3-fold at 60–120 min). The radiotracer was stable in the plasma and distributed well to all the tissues studied. The 60-min tumour/muscle ratios positively correlated with the percentage of *p*O_2_ values <5 mmHg (*r*=0.805, *P*=0.027) and carbogen breathing decreased [^18^F]FETA-derived radioactivity levels (*P*=0.028). In contrast, nitroreductase activity did not influence accumulation. Tumours were sufficiently visualised by PET imaging within 30–60 min. Higher fractional retention of [^18^F]FETA in HT1080/1-3C *vs* HT1080/26.6 tumours determined by dynamic PET imaging (*P*=0.05) reflected higher percentage of *p*O_2_ values <1 mmHg (*P*=0.023), lower vessel density (*P*=0.026), and higher radiobiological hypoxic fraction (*P*=0.008) of the HT1080/1-3C tumours. In conclusion, [^18^F]FETA shows hypoxia-dependent tumour retention and is, thus, a promising PET marker that warrants clinical evaluation.

Hypoxia occurs to a variable extent in most rodent and human solid tumours (Moulder and Rockwell, 1984; Höckel and Vaupel, 2001). It results from an inadequate and disorganised tumour vasculature and blood flow, and hence impaired oxygen delivery (Vaupel *et al*, 1989). Detection of hypoxia is important because hypoxia is a powerful trigger for gene expression and thus clonal selection of a more aggressive phenotype, for example, diminished apoptotic potential (Gräber *et al*, 1996; Koong *et al*, 2000). Of immediate clinical importance, hypoxia reduces local tumour control by external beam radiotherapy (Horsman and Overgaard, 1992) and predicts general treatment outcome, including metastatic potential and survival following radio/chemotherapy and surgery in a number of human cancers (Höckel *et al*, 1993; Brizel *et al*, 1996). Thus, detection of hypoxia could improve patient selection for therapy with bioreductive agents (Stratford and Workman, 1998), modified radiotherapy regimes such as accelerated radiotherapy with carbogen and nicotinamide ‘ARCON’ (Hoskin and Saunders, 1994), conformal radiotherapy, and hypoxia-targeted gene therapy (Dachs *et al*, 1997). Beyond oncology, potential applications exist for diagnosis of stroke, ischemic heart disease, peripheral vascular disease, arthritis, and anaerobic infection.

Studies with fine needle oxygen electrodes provided proof that low *p*O_2_ leads to poor outcome after treatment (Höckel *et al*, 1996; Brizel *et al*, 1997; Nordsmark and Overgaard, 2000; Fyles *et al*, 2002). Immunohistochemical parameters obtained by staining biopsy sections with antibodies to EF5, pimonidazole, hypoxia-inducible factor 1*α* (HIF-1*α*^3^), and carbonic anhydrase IX have also shown promise (Evans *et al*, 1996; Wykoff *et al*, 2000; Aebersold *et al*, 2001; Kaanders *et al*, 2002). Utility in a routine clinical setting would, however, favour a simple, convenient, noninvasive method for detecting tumour hypoxia that yields results, which correlate with clinical outcome. In this regard, a number of probes have been developed for imaging hypoxia by positron emission tomography (PET) and single-photon emission tomography in nuclear medicine. These include ^18^F-labelled 2-nitroimidazoles such as [^18^F]FMISO (Koh *et al*, 1995; Rasey *et al*, 1996), [^18^F]EF5 (Ziemer *et al*, 2003), [^18^F]FETNIM (Yang *et al*, 1995), [^18^F]FAZA (Sorger *et al*, 2003), and *N*-(2-([^18^F]fluoroethyl)-2-(2-nitroimidazol-1-yl)-acetamide ([^18^F]FETA) (Rasey *et al*, 1999); copper bis-thiosemi-carbazones such as [^60^Cu]ATSM (Fujibayashi *et al*, 1997; Dehdashti *et al*, 2003); technetium-based probes such as [^99m^Tc]HL-91 (Honess *et al*, 1998); and iodine-based probes such as [^123^I]IAZA (Mannan *et al*, 1991). Due to various limitations, none of these radiotracers have found their way into routine clinical use (Workman and Brown, 1981; Nunn *et al*, 1995; Rasey *et al*, 1999; Bentzen *et al*, 2002).

Of the numerous possibilities in nuclear medicine, appropriately labelled 2-nitroimidazole probes are particularly attractive for imaging tumour hypoxia (*p*O_2_ <10 mmHg (Höckel *et al*, 1996; Nordsmark *et al*, 1996); radiobiological hypoxia, *p*O_2_ <1 mmHg (Rasey *et al*, 1990)). A combination of pharmacological and physical properties that need to be considered as part of an ideal design goal include: (i) a nitro group with appropriate redox potential (E_1/7_ of ∼−380 to −390 mV) for selective reduction and binding in hypoxic tumour cells; (ii) lipophilicity that is high enough to enable diffusion across cellular membranes to the site of metabolism (octanol–water partition coefficient of ∼⩾0.1 (Brown and Workman, 1980; Workman, 1982), but low enough (∼⩽2) to assure rapid systemic elimination and, hence, convenient imaging times (within 2 h); (iii) stability to hypoxia-independent degradation; and (iv) high photon flux (and low energy) to assure high detection sensitivity and spatial image resolution. The last property would favour ^18^F-based PET radiotracers. In this manuscript we report on preclinical studies that underpin the development of [^18^F]FETA ([^18^F]fluoroetanidazole), an extremely promising hypoxia probe for imaging by PET. We demonstrate for the first time in mouse models of cancer that the retention of [^18^F]FETA-derived radioactivity correlates with *p*O_2_ and radiation sensitivity.

## MATERIALS AND METHODS

### Radiosynthesis of [^18^F]FETA

**Figure 1:**
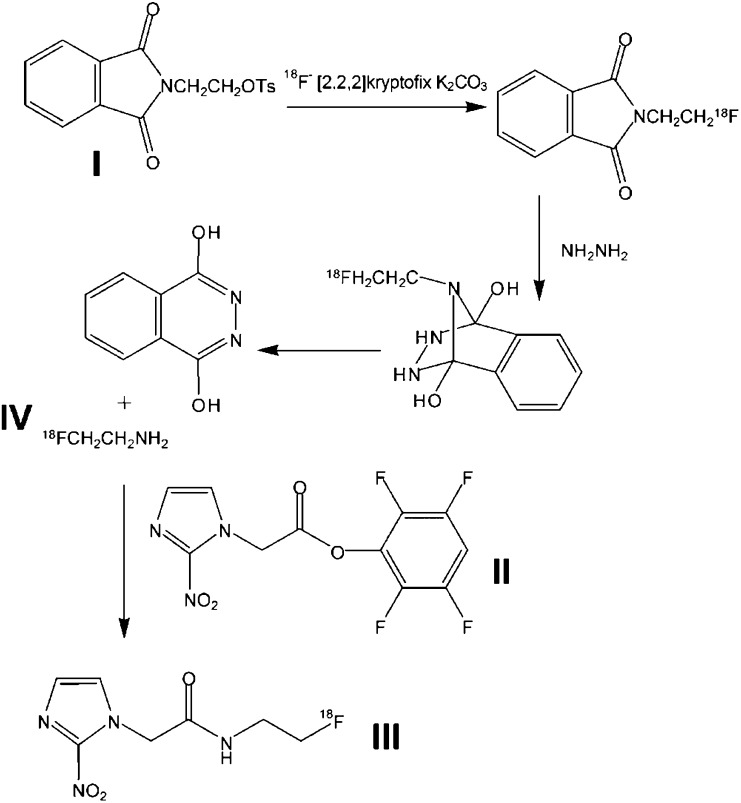
Radiochemical synthesis of [^18^F]FETA. I, II, III, and IV represent *N*-[2-(toluene-4-sulphonyloxy)-ethyl]-phthalimide, 2,3,5,6-Tetrafluorophenyl 2-(2-nitroimidazol-1-yl) acetate, [^18^F]Fluoroetanidazole, and [^18^F]Fluoroethylamine.

Unless otherwise noted, all reagents, including anhydrous solvents, were obtained from Sigma-Aldrich (Poole, UK). The synthesis of [^18^F]FETA was based on the method of Tewson (1997), with some modifications. *N*-[2-(toluene-4-sulphonyloxy)-ethyl]-phthalimide (I in Figure 1

) was prepared according to the literature method (Tewson, 1997). The precursor, 2,3,5,6-Tetrafluorophenyl 2-(2-nitroimidazol-1-yl) acetate, (II) was prepared by a novel method, published separately (Wilson, 2003). [^18^F]fluoride was prepared by the ^18^O(*p,n*)^18^F reaction using H_2_^18^O enriched water (Ruth and Wolf, 1979). A measure of 250 *μ*l 0.1 M K_2_CO_3_ and 3 mg [2,2,2]Kryptofix in 500 *μ*l acetonitrile were added to the [^18^F]fluoride. The fluoride was dried in a glassy carbon vessel at 125°C, under N_2_, for 35 min (with two further additions of 500 *μ*l acetonitrile), before cooling. In total, 15 mg of compound (I) was dissolved in 500 *μ*l acetonitrile. This was added to the reaction vessel, which was sealed at 100°C for 10 min before evaporation of the acetonitrile. A measure of 75 *μ*l hydrazine hydrate was added to the reaction vessel. [^18^F]fluoroethylamine (IV) was distilled from the reaction vessel (75°C, N_2_ flow ∼3 ml min^−1^) into a vial containing 4 mg precursor (II) in 400 *μ*l of ice cold acetonitrile. The vial was sealed and stirred for 30 min at room temperature. The resulting product was purified by high-performance liquid chromatography (HPLC) (Waters *μ*-Bondapak C_18_ column) and eluted with water (Baxter Healthcare Ltd, Compton, UK; 3 ml min^−1^). The radioactive product having the same retention time as fluoroetanidazole was collected (reference material synthesised according to Tewson, 1997). The product was evaporated to dryness and taken up in 0.9% saline (Phoenix Pharma Ltd, Gloucester, UK) for injection. The (manual) preparation time for [^18^F]FETA (synthesis, purification, formulation) from the end of bombardment was approximately 3 h.

### Lipophilicity measurements

The lipophilicity of [^18^F]FETA was determined by measuring the octanol–water partition coefficient. For this, 80–100 *μ*Ci (2.96–3.70 MBq) of [^18^F]FETA was dissolved in distilled H_2_O to a final volume of 0.5 ml and 0.5 ml of octan-1-ol (BDH Chemicals Ltd, Poole, UK) was added. The resulting mixture was processed in an orbital shaker (Sanyo Gallenkamp PLC, Loughborough, UK) for 10 min at 300 rpm and subsequently centrifuged for 30 min (12 000 rpm; Rotina 35R, Hettich Zentrifugen, Tuttlingen, Germany). Aliquots (200 *μ*l) of the resulting top layer (representing [^18^F]FETA dissolved in octanol) and bottom layer ([^18^F]FETA dissolved in H_2_O) were taken and the radioactivity in these samples was measured using a Cobra II Auto-Gamma counter (Packard Instruments, Meriden, CT, USA). Six octanol–H_2_O mixtures were analysed and the complete experiment was repeated once. The octanol–water partition coefficient was calculated by dividing the octanol-containing radioactivity by the water-containing radioactivity.

### Cellular uptake of [^18^F]FETA

The inhibitory effect of oxygen on [^18^F]FETA uptake by cells was studied *in vitro* in RIF-1 tumour cells. Uptake was defined as the transport of radiotracer into cells and its specific binding to cellular macromolecules (and glutathione). The cells were cultured (5% CO_2_ incubator at 37°C) in T75 flasks in RPMI growth medium (Life Technologies, Strathclyde, UK) supplemented with 10% foetal bovine serum (Sigma) and antibiotics. Exponentially growing cells were harvested, dissolved in phosphate-buffered saline (PBS) and placed in a custom-made hypoxic chamber (Aboagye *et al*, 1997a), which was positioned in a 37°C shaking water bath and incubated with pure nitrogen (O_2_ <20 ppm; BOC Gases, Guildford, UK) or with air as control, each flowing at 2 l min^−1^. After pregassing for 8 min, the cells were incubated with 80–100 *μ*Ci (2.96–3.7 MBq) of [^18^F]FETA. No correction for the total amount of stable etanidazole in the reaction mixture was made between experiments. Immediately after addition of the radioactivity, and at 15, 30, 45, 60, 90, and 120 min, the cells were harvested and centrifuged (5000 rpm for 5 min) to obtain a pellet. The pellet was washed twice with PBS (5 ml) and centrifuged as above. The cell number in the resulting pellet was determined by counting cells that excluded trypan blue. The total radioactivity added to each hypoxic chamber and the cell-bound radioactivity were measured on a Cobra II Auto-Gamma counter (Packard Instruments, Meriden, Connecticut, SA, USA) to determine the degree of radioactivity binding. Percentage of bound radioactivity was calculated as (bound radioactivity in 10^6^ cells × 100)/total radioactivity. These experiments were carried out in triplicate and repeated independently three times.

### Animals and tumours

The mice used in this work were obtained from Harlan UK Ltd (Bicester, UK). To obtain tumours, 2 × 10^5^–2 × 10^6^ tumour cells harvested from exponentially growing *in vitro* cultures were injected into the dorsum subcutis of normal C3H/Hej and Balb/c mice (for RIF-1 mouse fibrosarcoma and EMT6 mouse mammary carcinoma tumours, respectively) or Balb/c nude mice (for HT1080 human fibrosarcoma and MCF-7 human mammary carcinoma tumours). In the case of the HT1080 tumours, two subclones (1-3C, 26.6) with different capacities for producing vascular endothelial growth factor (VEFG) were studied. As published recently by our group, the 26.6-subclone produces two- to four-fold more VEGF than the 1-3C-subclone (Collingridge *et al*, 2002). To obtain MCF7 tumours, a 60-day slow release 17-*β*-oestradiol pellet (Innovative Research of America, Sarasota, FL, USA; 0.72 mg pellet^−1^) was inserted subcutaneously on the contralateral flank of the mouse at the time of inoculation of the tumour cells to maintain blood levels of 300–400 pg ml^−1^. All the animal work was performed by licensed investigators in accordance with the United Kingdom's ‘Guidance on the Operation of Animals (Scientific Procedures) Act 1986’ (HMSO, London, UK, 1990) and in full compliance with government regulations and the UKCCCR guidelines on animal welfare in experimental neoplasia (Workman *et al*, 1998). Tumours were selected for experiments when they had reached 5–8 mm in diameter (100–300 mg). In order to obtain comparable results, all biodistribution studies, PET imaging studies, and *p*O_2_ measurements were performed in anaesthetised mice. For that, a general neuropleptanalgesia comprising of a mixture of fentanyl citrate/fluanisone (Janssen-Cilag Ltd, Saunderton, UK; 0.79 and 25 mg kg^−1^ respectively) and midazolam hydrochloride (Phoenix Pharma, Ltd, Gloucester, UK; 12.5 mg kg^−1^) was injected i.p. 5 min before the start of the experiments. Irradiation of mice, in studies to determine radiobiological hypoxic fraction, were performed in unanaesthetised mice.

### Biodistribution of [^18^F]FETA

Anaesthetised tumour-bearing mice were injected intravenously via the lateral tail vein with 100 *μ*l of [^18^F]FETA (10–100 *μ*Ci; 0.37–3. 7 MBq). Mice were killed by exsanguination via cardiac puncture 60 min after injection of the radiotracer. Aliquots of heparinised blood were rapidly centrifuged (2000 **g** for 5 min) to obtain plasma. The radioactivity contained in tumour, liver, kidney, spleen, lungs, heart, small intestines, brain, muscle, bone, blood, plasma, faeces, and urine was determined in a Cobra II Auto-Gamma counter and expressed as % ID g^−1^ (percentage of injected dose per gram of tissue). A minimum of six mice was used for each tumour model.

Biodistribution studies were also carried out in mice following modulation of tumour hypoxia. In RIF-1 tumour-bearing mice, the degree of tumour hypoxia was modulated by administration of carbogen gas (95% O_2_, 5% CO_2_), which is known to decrease tumour hypoxia (Powell *et al*, 1999). There are a number of reports in the literature demonstrating an increase in oxygenation or perfusion in RIF-1 tumours under carbogen breathing. These studies include direct *p*O_2_ measurements, as well as magnetic resonance and ^86^Rb extraction methods in RIF-1 tumours implanted in the leg (Hasegawa *et al*, 1987; Song *et al*, 1987; Ilangovan *et al*, 2002) or flank (Helmer *et al*, 1998; Dardzinski and Sotak 1994; Honess and Bleehen, 1995). Carbogen gas was delivered via a nose cone at 2.0 l min^−1^, starting 2 min before radiotracer injection and for 60 min after radiotracer injection (*n*=5). Untreated RIF-1 tumour-bearing mice served as controls (*n*=8). Mice were injected with [^18^F]FETA and killed at 60 min postinjection (p.i.), as described above. Successful modulation of tumour *p*O_2_ was verified by means of OxyLite probes as described below.

### Small animal [^18^F]FETA-PET imaging

In parallel with the 60-min biodistribution studies above, the time course of [^18^F]FETA distribution was studied *in vivo* in tumour-bearing mice by means of a second-generation dedicated small animal PET scanner (Quad-HIDAC; Oxford Positron Systems, Weston-on-the-Green, UK (Jeavons *et al*, 1999)). Mice were anaesthetised as described above and placed prone on a thermostatically controlled bed within the scanner. All anaesthetised animals (in the PET imaging, biodistribution and OxyLite *p*O_2_ studies) were placed on thermal platforms to maintain their body temperature at ∼37°C. Dynamic PET scans were acquired over 60 min after intravenous (i.v.) injection of 40–100 *μ*Ci (1.48–3.7 MBq) of [^18^F]FETA via a tail vein cannula. The imaging and image-data processing protocol has been recently described in detail elsewhere (Barthel *et al*, 2003). In brief, data were acquired in list mode format and image reconstruction was performed by filtered back-projection using a 2D Hamming filter (cutoff 0.6). The image data sets obtained were transferred to a SUN workstation (Ultra 10; SUN Microsystems, Santa Clara, CA, USA) and visualised using the ANALYZE software (Version 5.0; Biomedical Imaging Resource, Mayo Clinic, Rochester, NY, USA). Region(s) of interest (ROIs) were defined for tumour and heart cavity (to provide information on the delivered radioactivity) on three to six transverse planes for tumour and three planes for heart cavity. Time *vs* radioactivity curve(s) (TACs) from the ROIs were averaged for each tumour and normalised to the integral of the heart cavity TAC. Altogether, four HT1080/1-3C and three HT1080/26.6 tumour-bearing mice were scanned.

### *In vivo* metabolism of [^18^F]FETA

Plasma, urine, liver, gall bladder, muscle, and tumour samples obtained from RIF-1 tumour-bearing C3H/Hej mice were assessed for putative [^18^F]FETA metabolites by HPLC. For this, plasma samples (100 *μ*l) were deproteinated by adding 2.0 ml ice-cold methanol and centrifuged (3000 **g**, 10 min, 4°C). Liver, muscle, and tumour samples were cut into small pieces and homogenised in 2.0 ml of ice-cold methanol using an Ultra-Thurrax homogeniser (IKA, Staufen, Germany) and the resultant homogenate was centrifuged (3000 g, 10 min, 4°C). The supernatants from plasma and tissues were evaporated to dryness in a rotary evaporator (50°C) under vacuum, reconstituted in 1.5 ml of mobile phase (methanol/H_2_O (20/80% v v^−1^)), centrifuged and filtered (0.2 *μ*m). Unlike plasma and tissue samples, urine and gall bladder samples were diluted with 1.5 ml of mobile phase and clarified by filtration (0.2 *μ*m). Aliquots of each filtrate (1.0 ml) and of the injected dose solution diluted in 1.5 ml of mobile phase were analysed by HPLC. The samples were separated on a C_18_ *μ*Bondapak column (7.8 × 300 mm, size 10 *μ*m; Waters, Milford, MA, USA) that was eluted with the aforementioned mobile phase at a flow rate of 2.0 ml min^−1^. The radioactivity of the eluents was monitored. Peak areas were integrated and corrected for physical decay and background radioactivity.

### Direct measurement of *p*O_2_

Oxygenation in the established tumour models was measured using the OxyLite *p*O_2_ system (Oxford Optronix Ltd, Oxford, UK) (Young *et al*, 1996; Collingridge *et al*, 1997; Seddon *et al*, 2001). The OxyLite probes measure *p*O_2_ by using a fluorescence quenching technique (Young *et al*, 1996). These experiments were performed under general neuroleptanalgesia. The animals were placed on a thermal blanket (37°C) and a maximum of four (depending on the tumour size) oxygen probes were simultaneously implanted into the mouse tumours. The probes were moved forward manually ∼0.2 cm every 10 min (total measurement time 30–50 min), which resulted in 45–125 *p*O_2_ measurements per tumour. All oxygen measurements were postcalibrated to account for tumour temperature, as previously reported (Young *et al*, 1996; Collingridge *et al*, 1997).

### Measurement of vessel density

In parallel studies in HT1080 (grown from 1-3C and 26.6 subclones) tumour-bearing mice, tumours were excised for histological examination of vessel density. For this, each of seven 1-3C and 26.6 tumours were excised, fixed in formalin, embedded in paraffin, and cut into 5.0 *μ*m sections. Adjacent sections were stained with haematoxylin–eosin (H&E). Sections from three different regions of each tumour, separated by at least 1.0 mm, were used for the analyses. Vessels were counted in five randomly selected fields of view (0.23 mm^2^) per section using a BX51 Olympus microscope (Olympus Optical, Tokyo, Japan) at × 200 magnification.

### Determination of radiobiological hypoxic fraction

In addition to the *p*O_2_ measurements, radiobiological hypoxia was evaluated for the 26.6. and 1-3C subclones of the HT1080 tumours by performing clonogenic assays. Clonogenic cell survival, which is still regarded as gold standard for measuring tumour hypoxia (Rockwell and Moulder, 1990), was determined using an established protocol (Collingridge and Rockwell, 2000). In brief, the tumour-bearing mice were irradiated with 5, 15, or 25 Gy (mean dose rate=4.3 Gy min^−1^) using an IBL 637 irradiator (CIS bio international, Gif/Yvette Cedex, France). One group of mice breathed air during the irradiation; the other group comprised of mice killed 10 min before the irradiation (*n*=12 per group). Immediately following irradiation, the tumours were excised, minced, and digested for 45 min in a 37°C shaking water bath with a 10 ml solution of foetal bovine serum-free RPMI growth medium containing 2 mg ml^−1^ collagenase type IV (Sigma, Poole, UK) and 0.2 mg ml^−1^ DNAse I (Sigma). After centrifugation, cell density was determined by trypan blue exclusion and defined numbers of cells were plated in Petri dishes containing complete growth medium. The dishes were incubated at 37°C in a humidified incubator in an atmosphere of 95% air/5% CO_2_ for 10–14 days. The resulting colonies were fixed and stained with crystal violet made up in 70% ethanol and subsequently counted. The survival fraction was calculated by dividing the plating efficiency of the irradiated mice by that of unirradiated control mice (*n*=4). Using these survival curves, radiobiological hypoxia fraction was calculated from the vertical displacement of the aerobic curve from the anoxic curve according to the equation log_hypoxia fraction_=log_aerobic survival fraction_−log_anoxic survival fraction_ (Moulder and Rockwell, 1984). The determination of hypoxic fraction in this way is only possible if the survival curves are parallel. Pooled tumours from four animals were used for each irradiation level and tumour type.

### Nitroreductase activity

Tumour retention of an ideal radiolabelled 2-nitroimidazole should depend solely on intratumoural oxygenation levels, and not on the levels of nitroreductase activities. In this regard, we investigated the influence of tumour nitroreductase activities on [^18^F]FETA tumour retention. The activities of cytochrome *P*450 reductase, cytochrome *b*_*5*_ reductase, and DT-diaphorase were determined in excised, snap-frozen tumours using a standard cytochrome *c* assay, as previously described by Sharp *et al* (2000). Briefly, enzyme activities were determined on a UV-spectrophotometer (BioMate 5; Thermo Spectronic, Cambridge, UK) by measuring the rate of reduction of cytochrome *c* at 550 nm (Walton *et al*, 1991). Tumour samples were thawed and homogenised in an equivalent volume of 50 mM Tris – 150 mM KCl–HCl buffer (pH 7.4). The homogenates were centrifuged (10 000 rpm for 30 min, 4°C) and the resulting supernatant, which represented the S9 fraction, was taken for the further analysis. The protein content of the S9 fractions was determined using a commercial kit (BCA protein assay kit; Pierce, Rockford, IL, USA). Samples of the S9 fraction (5–30 *μ*l) were added to 1 ml of a reaction mixture containing 50 mM (final concentration) Tris HCl buffer (pH 7.4), 20 *μ*M menadione (Sigma) as initial electron acceptor, 70 *μ*M cytochrome *c* (Sigma) as terminal electron acceptor, 200 *μ*M NADH (Sigma) as electron donor, and 1.4 mg ml^−1^ bovine albumin fraction V (Sigma). The solutions were prewarmed at 37°C and the experiment was performed with or without 10 *μ*M dicumarol (Sigma), an established inhibitor of DT-diaphorase (Hollander and Ernster, 1975). DT-diaphorase activity was taken as dicumarol-inhibitable activity and expressed as nmol of cytochrome *c* reduced per minute per mg of protein. The activities of cytochrome *P*450 reductase and cytochrome *b*_*5*_ reductase were determined as above, but without menadione/dicumarol, and with 200 *μ*M NADPH (Sigma) instead of NADH in the case of cytochrome *P*450 reductase assay (Fitzsimmons *et al*, 1996; Winski *et al*, 1998).

### Statistics

Statistical analyses were performed using the software SPSS for Windows, version 10.0.7 (SPSS Inc., Chicago, IL, USA). Differences in *p*O_2_ data, *ex vivo* [^18^F]FETA biodistribution, and nitroreductase activities were tested for significance using analysis of variance (ANOVA). Two-sided Student's *t*-test for independent samples was employed to test for differences in [^18^F]FETA uptake *in vitro* under normoxic and hypoxic conditions, *p*O_2_ values, and *ex vivo* [^18^F]FETA retention in RIF-1 tumours treated with carbogen *vs* untreated controls, as well as differences between vessel density, clonogenic survival, and fractional radiotracer retention obtained from dynamic PET scanning in 26.6 and 1-3C subclones of HT1080 tumours. Associations between [^18^F]FETA tumour retention, *p*O_2_ values, and nitroreductase activities in the established tumour models were tested for correlations by means of linear regression analysis. *p*O_2_ values obtained from the OxyLite measurements were expressed as median values for all investigated mouse tumours. Survival fractions from clonogenic assays were calculated relative to the corresponding plating efficiency and are given as mean±1 s.e.m. Survival curves were fitted by regression analysis and compared at the midpoints of the curves. Unless stated, data are expressed as mean±1 s.e.m. The *P*-values of ⩽0.05 were considered significant.

## RESULTS

### Radiochemistry and *in vitro* characteristics of [^18^F]FETA

Beginning with 30–50 mCi (1.11–1.85 GBq) [^18^F]fluoride, [^18^F]FETA yields were typically 0.6–3.0 mCi (22–111 MBq), which represents a final (decay corrected to end of bombardment) radiochemical yield of approximately 10–20%. High-performance liquid chromatography analyses of the [^18^F]FETA dose solutions were performed 60 min after preparation of the radiotracer. [^18^F]FETA eluted at ∼9.5 min. The radiochemical purity was 94.6±1.5% (*n*=4). The lipophilicity of [^18^F]FETA expressed as octanol–water partition coefficient was determined to be 0.16±0.01 (*n*=12).

**Figure 2:**
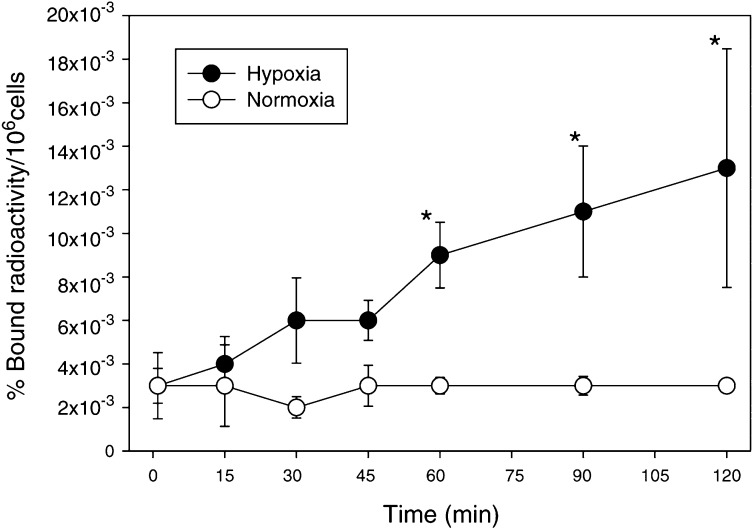
*In vitro* binding of [^18^F]FETA to RIF-1 cells under hypoxic (-•-) and normoxic (-○-) conditions. The cells were incubated with [^18^F]FETA under nitrogen gas or air for 0–120 min and washed to remove unbound radioactivity. The percentage of bound radioactivity was calculated as (bound radioactivity in 10^6^ cells × 100)/total radioactivity. Data are mean±s.e.m. (*n*=3), ^*^P⩽0.05.

We studied the uptake of [^18^F]FETA in air *vs* nitrogen in RIF-1 cells. The uptake of the radiotracer by normoxic cells was very low. In contrast, there was a rapid time-dependent linear increase of [^18^F]FETA uptake by RIF-1 cells under nitrogen, which was 3.0- and 4.3-fold as compared to the normoxia values at 60 and 120 min p.i., respectively. In this model system the hypoxia-dependent increase in [^18^F]FETA uptake reached significance after 60 min (Figure 2

).

### *In vivo* tissue distribution and metabolism of [^18^F]FETA

**Figure 3:**
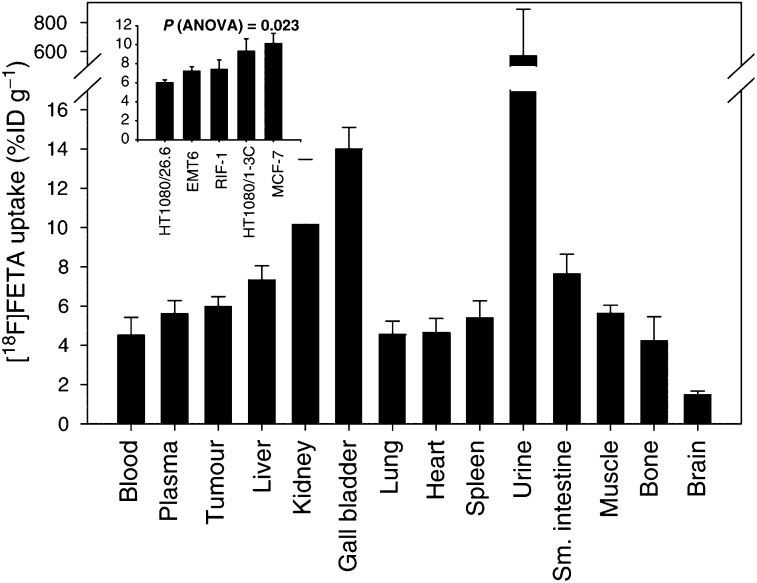
Tumour and normal tissue distribution of [^18^F]FETA at 1 h p.i. in HT1080/26.6 tumour-bearing male Balb/c nu/nu mice (*n*=10). Inset: radiotracer uptake in the different tumour types (*n*=5–10). Data are mean % ID g^−1^±s.e.m.

The normal tissue retention of the radiotracer at 60 min postinjection was low (<5% ID g^−1^) in lung, heart, brain, and bone; intermediate (5–8% ID g^−1^) in plasma, liver, spleen, small intestines, and muscle; and high (>8% ID g^−1^) in kidney, bile, and urine (Figure 3

). The 60-min tumour retention of [^18^F]FETA ranged from 6 to 10% ID g^−1^ for the different xenografts (*P*=0.023; one-factorial ANOVA; Figure 3 inset). Other than differences in tumour retention, no significant differences in radiotracer accumulation were found for normal tissues of the different mouse strains (data not shown).

**Figure 4:**
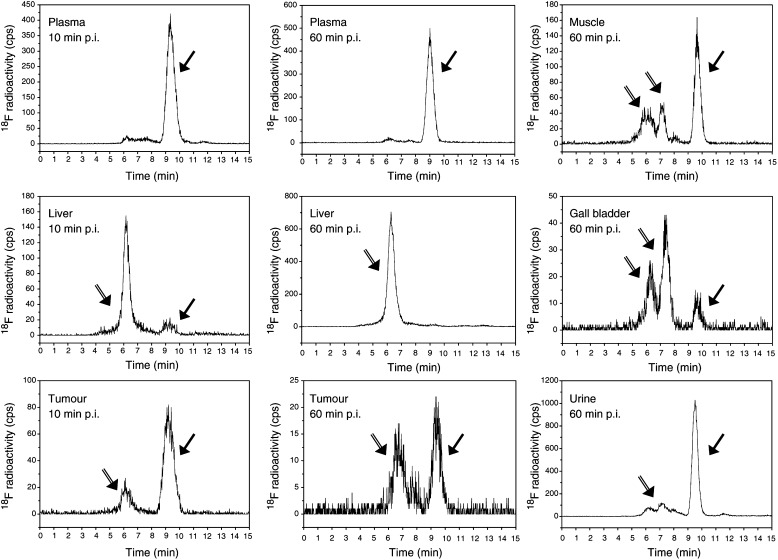
Reversed-phase high-performance liquid chromatograms of [^18^F]FETA and its putative metabolites at an early time-point (10 min p.i.) in plasma (**A**), liver (**D**), and tumour (**G**), and a late time-point (60 min p.i.) in plasma (**B**), muscle (**C**), liver (**E**), gall bladder (**F**), tumour (**H**), and urine (**I**). Filled arrows are parent [^18^F]FETA compound, and double-lined arrows are metabolites.

Radiochromatograms of plasma, liver, tumour, muscle, gall bladder, and urine that were obtained at 10 (plasma, liver, and tumour) and 60 min (all tissues and body fluids) after the administration of [^18^F]FETA are shown in Figure 4

. As with the analysis of the dose solution, the parent compound ([^18^F]FETA) eluted at a retention time of ∼9.5 min. Parent [^18^F]FETA comprised 88% of the total radioactivity in plasma at 10 min p.i. In tumour, a minor radioactive peak at a retention time of ∼6 min that comprised 18% of the total radioactivity was detected at 10 min p.i., whereas most of the radioactivity (92%) in the liver was associated with the metabolite(s) eluting at ∼6 min. At 60 min p.i., metabolite peaks with retention times of ∼6 to 7 min were detected in the plasma (7% of total radioactivity), muscle (50%), liver (99%), bile (82%), tumour (52%), and urine (26%) (Figure 4). These HPLC data represent profiles of soluble parent and metabolites. The acid-insoluble fraction obtained from PCA extraction (representing bound radioactivity) was 69.2% for tumour and 63.7% for liver tissues at 60 min p.i.

### [^18^F]FETA tumour retention correlates with *p*O_2_ but not nitroreductase activity

Various threshold *p*O_2_ values (<1, 2.5, 5, and 10 mmHg) have been used to describe the degree of hypoxia in tissues. In the tumour xenografts used here, the relative frequency of *p*O_2_ values <1.0 mmHg as determined by the OxyLite method ranged from 28±13 to 93±9% (*P*=0.008; one-factorial ANOVA). The relative frequency of *p*O_2_ values <2.5 mmHg ranged from 51±13 to 100±0% (*P*=0.057). In HT1080 tumours, the relative frequency of hypoxic *p*O_2_ values was significantly higher for the 1-3C subclone compared to the 26.6 subclone at a cutoff *p*O_2_ of 1.0 mmHg (93±9 *vs* 55±15%; *P*=0.023) and 2.5 mmHg (100±0 *vs* 66±16%; *P*=0.045).

**Figure 5:**
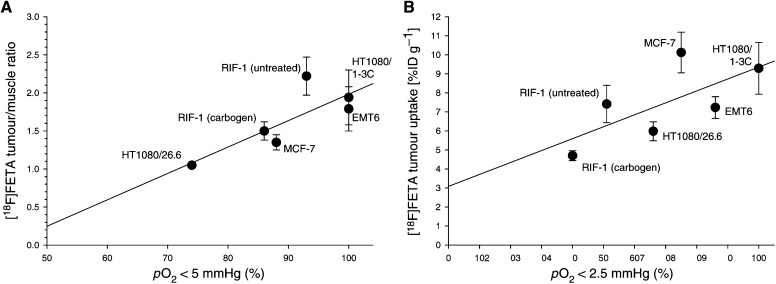
Association between tumour oxygenation as measured by OxyLite probes and [^18^F]FETA tumour uptake determined by *ex vivo* biodistribution studies. (**A**) Relative [^18^F]FETA uptake expressed as tumour-to-muscle ratio *vs* relative frequency of *p*O_2_ values <5 mmHg. (**B**) [^18^F]FETA uptake *vs* relative frequency of *p*O_2_ values <2.5 mmHg.

For all the tumour models, the relative [^18^F]FETA retention, expressed as tumour-to-muscle ratio, was positively correlated with the relative frequency of *p*O_2_ values <5 mmHg. This correlation was found to be linear (*y*=0.04*x*–1.5, *r*=0.805) and significant (*P*=0.027; Figure 5A

). A trend towards a significant positive correlation was also observed for the association between tumour [^18^F]FETA retention and relative frequency of *p*O_2_ values <2.5 mmHg (*r*=0.691, *P*=0.064; Figure 5B). Of interest, [^18^F]FETA radiotracer retention at 60 min p.i. was higher in the less VEGF-producing HT1080/1-3C subclone compared to the 26.6 subclone (9.29±1.36 *vs* 5.98±0.50% ID g^−1^; *P*=0.015). In addition, tumour/muscle ratios obtained from the [^18^F]FETA biodistribution studies were significantly higher for the 1-3C subclone as compared to the 26.6 subclone (1.94±0.36 *vs* 1.05±0.03, *P*<0.001).

The activities of cytochrome *P*450 reductase, cytochrome *b5* reductase, and DT-diaphorase for the different tumours are given in Table 1

**Table 1:** Nitroreductase activities in established tumours

**Tumour type**	**Cytochrome *P*450 reductase (nmol min^−1^ mg^−1^)**	**Cytochrome *b5* reductase (nmol min^−1^ mg^−1^)**	**DT-diaphorase (nmol min^−1^ mg^−1^)**
RIF-1	2.21±0.18	50.0±1.8	1.38±0.23
HT1080/26.6	3.39±0.36	36.8±1.4	0.93±0.01
HT1080/1-3C	4.15±0.34	35.3±2.5	0.21±0.05
EMT6	2.79±0.37	52.2±1.9	0.62±0.15
MCF-7	2.01±0.17	20.8±1.0	0.00±0.00^a^
*P* (ANOVA)	0.001	<0.001	<0.001

Enzyme activities were determined spectroscopically by measuring reduction of cytochrome *c* at 550 nm, as described in Materials and Methods. Units are nmol of cytochrome *c* reduced per minute per mg of sample protein and are given as mean±s.e.m. (*n*=5–7).

a No activity detected within effective range of assay (lower limit of detection=0.05 nmol min^−1^ mg^−1^).

. There were significant intertumour differences (one-factorial ANOVA) in enzyme activity. The range of these values, however, were ⩽2.5 fold for all the reductive enzymes tested (Table 1). There were no correlations between tumour enzyme profiles (cytochrome *P*450 reductase, cytochrome *b5* reductase, and DT-diaphorase) and *ex vivo* [^18^F]FETA tumour retention or tumour *p*O_2_ obtained from OyxLite measurements.

### Acute changes in *p*O_2_ are detectable with [^18^F]FETA

The ability to detect acute changes in tumour oxygenation was investigated in RIF-1 tumours after application of a physiological modulator, for which the resultant effect is well documented. Carbogen breathing decreased [^18^F]FETA tumour retention to 4.70±0.26% ID g^−1^, compared to untreated controls (7.41±0.98% ID g^−1^; *P*=0.028). In tissues other than tumours, [^18^F]FETA retention was not altered by carbogen breathing. Administration of carbogen decreased the relative frequency of *p*O_2_ values <2.5 mmHg in comparison to untreated controls (40±11 *vs* 51±13%). However, these differences did not reach significance level (*P*=0.551).

### Small animal PET imaging with [^18^F]FETA

**Figure 6:**
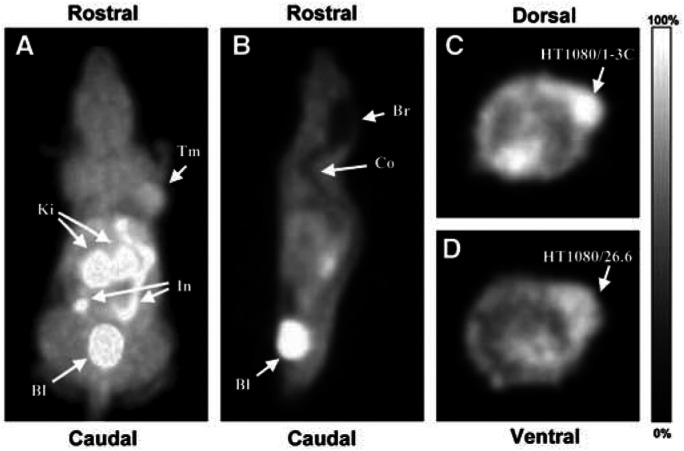
[^18^F]FETA-PET images of HT1080 tumour-bearing mice acquired on the small animal quad-HIDAC scanner (pixel size 0.5 × 0.5 × 0.5 mm^3^). (**A**) Three-dimensional (volume-rendered) image of an HT1080/1-3C tumour-bearing mouse (30–60 min p.i. summed) showing a dorsal view of the mouse. Here, pixel values are defined by the maximum voxel value in corresponding lines in the *z*-axis. Arrows point to tumour (Tm), Kidneys (Ki), small intestine (In), and urinary bladder (Bl). (**B**) Sagittal (0.5 mm) slice of 30–60 min p.i. summed [^18^F]FETA-PET images from the same mouse as in (**A**) at the midplane level, showing low radiotracer uptake in brain (Br) and spinal cord (Co), as well as high accumulation in urinary bladder. (**C**) Transverse (0.5 mm) slice of 30–60 min p.i. summed [^18^F]FETA-PET images from the same HT1080/1-3C tumour-bearing mouse as in (**A**) at the level of the maximal tumour diameter. (**D**) Corresponding transverse slice from an HT1080/26.6 tumour-bearing mouse, exhibiting lower tumour radiotracer uptake.

Whole body PET scanning was carried out in HT1080/26.6 (*n*=3) and HT1080/1-3C tumour-bearing mice (*n*=4). For illustration purposes, the 30–60 min summed images are presented (Figure 6

). In agreement with the biodistribution data determined by *ex vivo γ*-counting, the PET images revealed high [^18^F]FETA accumulation in tumour, kidney, urinary bladder, and proximal parts of the small intestine (Figure 6A). In contrast, radiotracer retention was low in brain and spinal cord (Figure 6B). Also in agreement with the biodistribution studies, the tumour retention of [^18^F]FETA was higher in all HT1080/1-3C tumours (Figure 6C) than in the HT1080/26.6 tumours (Figure 6D).

**Figure 7:**
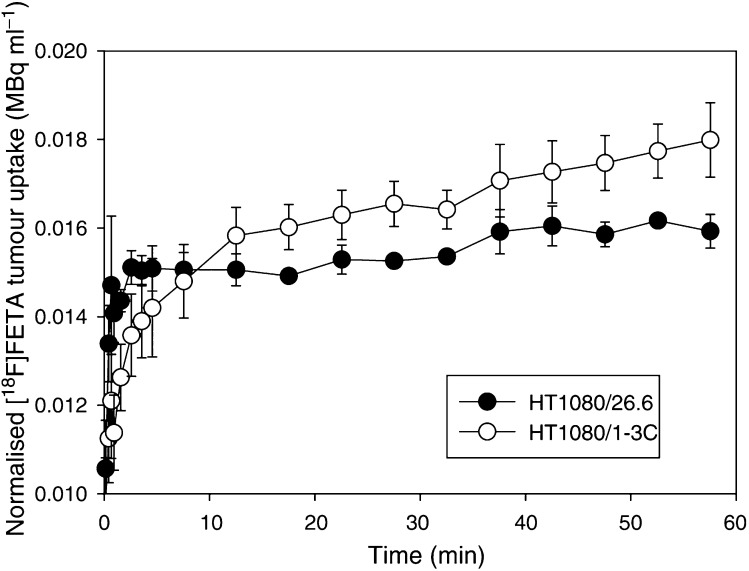
Tumour time–activity curves (TACs), normalised to the integral of the heart cavity TAC. The curves were obtained from region of interest analysis of dynamic [^18^F]FETA-PET images from HT1080/26.6 (-•-) and HT1080/1-3C (-○-) tumour-bearing mice. Data are mean±s.e.m. (*n*=3–4 per group).

By drawing ROIs over the whole tumour, TACs were obtained from the dynamic PET data sets (Figure 7

). HT1080/26.6 tumours were characterised by a rapid delivery of [^18^F]FETA up to 2 min p.i., followed by a plateau with a minimal increase of the TACs up to 60 min p.i. The delivery of the radiotracer into HT1080/1-3C tumours was comparatively slower, but retention was higher (Figure 7). The fractional retention of [^18^F]FETA in tumours (ratio of radioactivity at 60 min to that at 2 min) was significantly higher in the 1-3C tumours compared to 26.6 tumours (1.44±0.10 *vs* 1.10±0.01; *P*=0.05).

### Biological basis and implication of [^18^F]FETA retention in HT1080 tumours

**Figure 8:**
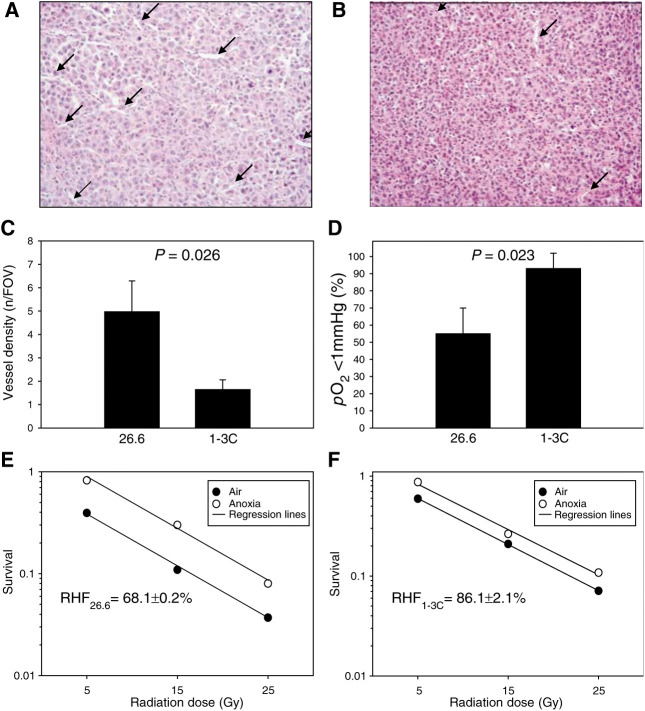
Vessel density, *p*O_2_, and radiation sensitivity of HT1080/26.6 and HT1080/1-3 tumours. (**A–B**) Typical 5 *μ*m H&E-stained histological sections of HT1080/26.6 (**A**) and HT1080/1-3C (**B**) tumours, with arrows pointing to vessels. (**C**) Summary data for vessel density depicting average number of vessels from five randomly selected fields of view (0.23 mm^2^; × 200 magnification) per section; three sections per tumour. (**D**) OxyLite *p*O_2_ measurements. (**E–F**) Radiation sensitivity determined in clonogenic assays and expressed as radiobiological hypoxic fraction of HT1080/26.6 (**E**) and HT1080/1.3C (**F**) tumours. Data are mean survival (*n*=4). RHF, radiobiological hypoxic fraction. There was a statistically significant difference between the RHF of 1-3C and 26.6 tumours (*P*=0.008).

In order to explain the differences in [^18^F]FETA retention between the different HT1080 tumour types, we compared the retention of [^18^F]FETA in the tumours to vessel density, oxygenation, and radiation sensitivity. The vessel density measured in histological sections of the HT1080 tumours was significantly lower for the 1-3C subclone, which contains less VEGF (Collingridge *et al*, 2002), compared to the 26.6 subclone (Figures 8A–C

). The lower vessel density in the 1-3C tumours was associated with a significantly higher frequency of *p*O_2_ values <1 mmHg (Figure 8D). Radiation sensitivity was determined for the two tumour types by measuring the fraction of clonogenic radiobiological hypoxic cells. The plating efficiency (mean±s.e.m.) of HT1080/1-3C and/26.6 tumours were 0.50±0.03 and 1.70±0.41%, respectively. Overall, disaggregation of the tumours yielded (irradiated *vs* control) 2 to 20 × 10^6^ cells *vs* 4 to 20 × 10^6^ cells for HT1080/1-3C and 1 to 11 × 10^6^ and 2 to 9 × 10^6^ cells for HT1080/26.6 cells, respectively. Figures 8E, F show that hypoxic fraction was significantly higher in HT1080/1-3C than in HT1080/26.6 tumours (86.1±2.1 *vs* 68.1±0.2%, *P*=0.008; Figures 8E, F).

## DISCUSSION

The partial pressure of oxygen has been directly determined in accessible tumours such as cervix tumours, head and neck tumours, and sarcomas using Eppendorf oxygen electrodes (Höckel *et al*, 1996; Brizel *et al*, 1997; Nordsmark and Overgaard, 2000; Fyles *et al*, 2002). Such studies provided proof that low *p*O_2_ (hypoxia) leads to poor outcome after cancer treatment. These findings, together with the well-known effect of hypoxia on radiation sensitivity as well as the development of hypoxia-modifying therapies, have strengthened the medical need for a noninvasive, clinically acceptable method of measuring hypoxia in patient tumours. Of the numerous possibilities available, the use of appropriately labelled 2-nitroimidazoles detectable by PET imaging is particularly attractive. In this paper, we have demonstrated that [^18^F]FETA is a suitable radiotracer for PET imaging of tumour hypoxia.

^18^F-labelled 1-(2-nitro-imidazolyl)-3-fluoro-2-propanol ([^18^F]fluoromisonidazole ([^18^F]FMISO) (Jerabek *et al*, 1986; Koh *et al*, 1995; Rasey *et al*, 1996)) is the most widely studied radiotracer for tumour hypoxia. This radiotracer has, however, failed to gain a wider acceptance for routine clinical application in a PET setting because of a number of limitations including: (i) slow accumulation in hypoxic tumours (scanning has been performed up to 5 h p.i. (Nunn *et al*, 1995; Bentzen *et al*, 2002)); (ii) low target-to-background contrast due to high nonspecific binding resulting from a relatively high lipophilicity (octanol/water partition coefficient=0.41 (Workman and Brown, 1981)); and (iii) significant non-oxygen-dependent metabolism (e.g. 50.0 and 36.2% of metabolites in plasma and urine, respectively, at 2 h p.i. (Rasey *et al*, 1999)). The choice of an ideal hypoxia marker would favour probes that have low lipophilicity and resistance to hypoxia-independent degradation; in general 2-nitroimidazoles with an amide side chain typified by etanidazole are more stable to nonhypoxic metabolism than those with a hydroxypropyl side chain such as misonidazole (Aboagye *et al*, 1998). [^18^F]FETA possesses these favourable characteristics. Its octanol–water partition coefficient, determined here to be 0.16, is higher than nonfluorinated etanidazole (0.046 (Brown and Workman, 1980)), but lower than that of [^18^F]FMISO. *In vitro*, [^18^F]FETA accumulated selectively in hypoxic, but not in aerobic RIF-1 cells (4.3-fold differential at 120 min p.i.), consistent with the presence of a 2-nitroimidazole moiety (with redox potentials (E_1/7_) around −380 to −390 mV (Adams *et al*, 1979; Brown and Workman, 1980; Workman and Brown, 1981). The large variation in uptake could be due to the differences in stable etanidazole levels in the reaction mixture.

[^18^F]FETA accumulated in all tissues analysed. The retention of the radiotracer at 60-min postinjection was relatively low (<5% ID g^−1^) in lung, heart, brain, and bone; intermediate (5–8% ID g^−1^) in plasma, liver, spleen, small intestines, and muscle; and high (>8% ID g^−1^) in kidney, bile, and urine. The highest [^18^F]FETA accumulation was found in the excretory organs. Good accumulation in tumours, 6–10% ID g^−1^, would suggest the possibility of achieving high tumour-to-background contrast in several tumour types, including breast, lung, head and neck, and brain tumours. Chromatographic analysis of plasma, urine, and tissues provided some explanation for the differences in tissue distribution. [^18^F]FETA was found to be stable in plasma (∼93% as parent compound at 60 min p.i.) and also excreted mainly as the unchanged drug in urine (74% at 60 min p.i.). The radiotracer was metabolised in liver to catabolite(s), which appeared to be eliminated predominantly via the hepatobiliary route.

Having shown that [^18^F]FETA is taken up into all tissues, we investigated whether differences in *p*O_2_ could be monitored with the radiotracer *in vivo*. Two validation scenarios were explored. In the first instance, the 60-min retention of [^18^F]FETA into all tumour types as determined by *γ*-counting was compared to measured *p*O_2_. Radiotracer retention positively correlated with the percentage of *p*O_2_ values <5 mmHg (*r*=0.805, *P*=0.027), indicating that [^18^F]FETA retention is related to *p*O_2_ levels. Using five different tumour types enabled a wide range of oxygenation levels to be achieved (the relative frequency of *p*O_2_ values <1.0 mmHg ranged from 28±13 to 93±9% (*P*=0.008; one-factorial ANOVA), and the relative frequency of *p*O_2_ values <2.5 mmHg in a range from 51±13 to 100±0% (*P*=0.057)). In general, the binding of nitroimidazoles requires the presence of nitroreductases, particularly cytochrome *P*450 reductase. We found no relationship between [^18^F]FETA and levels of the nitroreductases studied. Large differences in nitroreductase levels can confound measurement of hypoxia with 2-nitroimidazole-based probes; thus, our findings of a lack of significant differences in nitroreductase activities between the tumour types used is a relevant contribution to the interpretation of [^18^F]FETA data. The range of nitroreductase levels observed here, ⩽2.5-fold, is unlikely to influence radiotracer retention due to a square-root relationship between the binding of 2-nitroimidazoles and cytochrome *P*450 reductase levels (Joseph *et al*, 1994). Rasey *et al* (1999) have shown that the half-maximal inhibition of [^18^F]FETA binding by oxygen is fairly similar (up to two-fold) across cell lines *in vitro*. This observation may be explained by the small differences in nitroreductase levels between cell lines. In the second instance, we investigated the ability of [^18^F]FETA to detect acute changes in tumour oxygenation. Carbogen breathing decreased the 60-min tumour retention of [^18^F]FETA by 36%, in keeping with an 11% decrease of hypoxic *p*O_2_ values measured by OxyLite electrodes. This finding is similar to those reported previously for other 2-nitroimidazoles (Aboagye *et al*, 1997b, 1998; Rasey *et al*, 2000; Bentzen *et al*, 2002; Seddon *et al*, 2002) and demonstrates that [^18^F]FETA can be used to detect acute changes in *p*O_2_.

PET studies performed in tumour-bearing mice demonstrated the feasibility of imaging hypoxia *in vivo*. Qualitatively, the PET images were in agreement with the quantitative tissue radioactivity data determined by *γ*-counting. It was also possible to perform semiquantitation of the dynamic PET data by ROI analysis. Interestingly, the tumour model with higher VEGF-expression (HT1080/26.6) showed lower fractional retention of the radiotracer compared to that with the lower VEGF-expression (HT1080/1-3C) despite a higher delivery of [^18^F]FETA into the former. This finding is in contradiction to the current dogma of ‘hypoxia inducing VEGF through the expression of HIF-1*α*’ (Harris, 2002; Semenza, 2003) and, thus the more hypoxic tumours being those exhibiting higher levels of VEGF expression. This observation may be explained in part by the lower (∼10-fold half-maximal inhibition) oxygen levels required to effect binding of 2-nitroimidazoles to cellular macromolecules *vs* that required to induce VEGF production (Leith and Michelson, 1995); in this regard, both tumour models are sufficiently hypoxic to induce VEGF production. The findings were, however, corroborated by a higher vessel density (explaining the higher radiotracer delivery) and a higher *p*O_2_ (explaining the lower fractional retention) in the HT1080/26.6 model. The impact of these findings on radiation sensitivity was that the HT1080/26.6 tumours were more sensitive to radiation than the HT1080/1-3C tumours as demonstrated by a lower clonogenic radiobiological hypoxic fraction of the former. Since the *p*O_2_ (and radiobiological hypoxic fractions) of the human tumour models used in this study have not been previously reported in the literature, an assessment of this (as well as the radiobiological hypoxic fraction for the HT1080 tumours) was made; the high and low *p*O_2_ values obtained for EMT6 and RIF-1 tumours, respectively (Moulder and Rockwell, 1984), corroborate our measurements.

Since the PET studies required the use of anaesthesia for immobilisation, all other *in vivo* studies were performed under anaesthesia. It is conceivable that most anaesthesia will decrease clearance of radiotracers from the body and give rise to a systematic increase in hypoxia levels within tumours. This may explain the very high percentage of *p*O_2_ values <2.5 mmHg seen in our experiments. These conditions have, however, been successfully used in other hypoxia experiments in mice to demonstrate nitroimidazole retention in tumours (Aboagye *et al*, 1997b). In translating this work into clinical studies one needs to bear in mind the fact that patients would be un-anaesthetised. The level of oxygenation may therefore be higher than that measured for the mouse tumours.

In summary we have validated an advanced-generation 2-nitroimidazole marker of tumour hypoxia in the mouse. Our findings indicate that [^18^F]FETA has suitable physicochemical properties and is stable to nonhypoxic degradation *in vivo*. We have also demonstrated that the tumour retention of the radiotracer is related to *p*O_2_ status and radiobiological hypoxia. These properties, as well as the ability to image hypoxia (*p*O_2_ values at 1–10 mmHg) dynamically with PET at early time points are attractive features that support the clinical development of [^18^F]FETA.
